# Earlier Outbreak Detection—A Generic Model and Novel Methodology to Guide Earlier Detection Supported by Data From Low- and Mid-Income Countries

**DOI:** 10.3389/fpubh.2020.00452

**Published:** 2020-09-11

**Authors:** Lindsay Steele, Emma Orefuwa, Silvia Bino, Shepherd Roee Singer, Julius Lutwama, Petra Dickmann

**Affiliations:** ^1^New York City Department of Health and Mental Hygiene, New York, NY, United States; ^2^Connecting Organizations for Regional Disease Surveillance (CORDS), Lyon, France; ^3^Institute of Public Health, Southern European Center for Surveillance and Control of Infectious Diseases (SECID), Tirana, Albania; ^4^Ministry of Health Israel, Jerusalem, Israel; ^5^East African Integrated Disease Surveillance Network (EAIDSNet), Kampala, Uganda; ^6^Department of Anaesthesiology and Intensive Care, Jena University Hospital, Jena, Germany; ^7^Dickmann Risk Communication Drc|, London, United Kingdom

**Keywords:** detection, surveillance, earlier detection, community-based surveillance, risk communication, methodology, time-to-detection, infectious disease outbreaks

## Abstract

Infectious disease outbreaks can have significant impact on individual health, national economies, and social well-being. Through early detection of an infectious disease, the outbreak can be contained at the local level, thereby reducing adverse effects on populations. Significant time and funding have been invested to improve disease detection timeliness. However, current evaluation methods do not provide evidence-based suggestions or measurements on how to detect outbreaks earlier. Key conditions for earlier detection and their influencing factors remain unclear and unmeasured. Without clarity about conditions and influencing factors, attempts to improve disease detection remain *ad hoc* and unsystematic.

**Methods:** We developed a generic five-step disease detection model and a novel methodology to use for data collection, analysis, and interpretation. Data was collected in two workshops in Southeast Europe (*n* = 33 participants) and Southern and East Africa (*n* = 19 participants), representing mid- and low-income countries. Through systematic, qualitative, and quantitative data analyses, we identified key conditions for earlier detection and prioritized factors that influence them. As participants joined a workshop format and not an experimental setting, no ethics approval was required.

**Findings:** Our analyses suggest that governance is the most important condition for earlier detection in both regions. Facilitating factors for earlier detection are risk communication activities such as information sharing, communication, and collaboration activities. Impeding factors are lack of communication, coordination, and leadership.

**Interpretation:** Governance and risk communication are key influencers for earlier detection in both regions. However, inadequate technical capacity, commonly assumed to be a leading factor impeding early outbreak detection, was not found a leading factor. This insight may be used to pinpoint further improvement strategies.

## Article Summary

### Strengths

This article adds three innovations to the existing body of research:

- The authors propose a generic five-step disease detection model that structures the process of disease detection in order to make it generically applicable and thus comparable;
- They describe and apply a methodology to systematically collect and analyze data that provides qualitative insights into key conditions and influencing factors for earlier detection of infectious disease outbreaks using the generic disease detection model; and
- This article provides qualitative insights into conditions and influencing factors for earlier detection in low- and mid-income countries.

### Limitations

- The generic model and the earlier-detection methodology can complement the quantitative time-to-detection measurement by providing qualitative, systematic social information. Building on this evidence, interventions can be designed to target the most relevant factors and conditions to lead to earlier detection. However, the empirical implementation is as yet pending.

## Introduction

### Background

The outbreak of Ebola viral hemorrhagic fever disease in West Africa stressed the importance of functional and reliable health systems that enable early disease detection, rapid response, and sustainable recovery. Ebola was not confirmed until three months after the first case, during which the virus spread rapidly from Guinea to two of its neighboring countries (Liberia and Sierra Leone). One year after the beginning of the epidemic, the death toll reached over 11,000 people in six countries with estimated GDP losses for these three most affected countries totaling US$ 2.2 billion. (1) The Ebola outbreak of 2012–2014 was a poignant reminder that the timeliness of outbreak detection has a significant impact on morbidity and mortality, economies, and social and cultural well-being of populations.

In the aftermath of the 2003 outbreak of the severe acute respiratory syndrome (SARS), the World Health Organization (WHO) updated the International Health Regulations (IHR 2005) and called for all Member States to build and strengthen their capacities to prevent, detect, and respond to infectious disease outbreaks, especially for those diseases that have the potential of international spread. The IHR (2005) addressed modern threats by expanding the usual infectious disease notification requirements to include “all events potentially constituting a public health emergency of international concern (PHEIC) (2). Through this binding agreement, Member States are required to establish core capacities and mechanisms for rapid detection of public health risks, as well as the prompt risk assessment, notification, and response to these risks. Risk communication as a core capacity under the IHR plays an important role in the identification and assessment of risks, internal, and external communication of risks, the coordination of response, and the implementation of lessons learned (3).

The international spread of disease can cause global repercussions with impacts on social systems, e.g., health (morbidity, mortality), economics (trade, travel, and employment), and social and cultural implications (education, religious practices, and social gatherings). Beyond the biomedical spread of diseases, anxiety and social perceptions can amplify the negative impacts of the disease on societies. Global fear and anxiety during the 2003 SARS pandemic, along with WHO and Centers for Disease Control and Prevention (CDC) travel advisories, tight quarantine policies, port infection control measures and intensive media coverage of the disease, had significant negative impacts on both travel and business around the world, particularly in Asia (4). Outbreaks can lead to stigmatization of populations, such as during the H1N1 outbreak when Mexicans and other Latinos living in the U.S. were ostracized as carriers of disease (5), and during the recent Ebola epidemic where, upon returning to communities, many survivors faced stigma, rejection, violence, and blame and found their jobs lost and properties destroyed (6). There can also be impacts on local industry, such as with H5N1, which has severe negative impacts for village poultry farmers in many Asian countries (7).

### Surveillance Narrative: Early Detection

Past outbreaks demonstrate a strong correlation between timely detection and successful containment measures in the sense that early detection is a key determinant of successful disease management, and mitigating impacts on individuals' health, and countries' economies and social systems. To enable early detection, surveillance systems must be in place. Surveillance is a core capacity under the IHR (2005) that requires each Member State to have an event monitoring system and strengthened surveillance capabilities for rapid detection, prompt risk assessment, notification, and response to public health risks. According to Global Health Observatory data on the implementation status of IHR surveillance core capacity requirements, reporting countries achieved 85% of the IHR surveillance requirements by 2014 (8). While there are many infectious disease surveillance systems in place that are meeting IHR surveillance core capacity requirements, a key concern is whether these systems detect diseases early and what measures could be undertaken to enable *earlier* detection.

### Time-to-Detection (TTD)

Significant time and funding have been invested in improving timeliness of detection (9). However, efforts to improve surveillance systems have struggled to determine what factors contribute to abilities to detect earlier. Currently, monitoring and evaluation follow a quantitative method that retrospectively measures the time (in days), “time-to-detection” (TTD), to discover and recognize an initial event as an outbreak (10, 11). This quantitative measure serves as a benchmark for monitoring infectious disease surveillance systems; however, it does not provide guidance on how to enable surveillance to detect earlier. Key influencing factors that enable or hinder early detection remain unclear and unmeasured. Without clarity about the influencing factors and measures to monitor and evaluate these factors, attempts to improve surveillance remain *ad hoc* and unsystematic. A recent systematic review of drivers of earlier detection revealed that there is little evidence about factors that influence earlier detection (12).

### Risk Communication

Current efforts lay great emphasis on technologies for improving early detection, with inadequate attention to governance and the role of awareness—both in the community and among health professionals—of the potential risk posed by infectious diseases, especially in the endemic settings of low- to mid-income countries. Thus, risk communication would be a central tool in addressing these shortfalls. Current research in the field of risk communication as a core capacity under IHR (2005) has suggested an evaluation framework for earlier detection, faster response, smoother coordination, and smarter legacy that can serve as a proxy for a better understanding of the drivers of earlier detection (13).

Risk communication, in this usage, is a horizontal activity in the process of infectious disease management that supports and informs core capacities such as surveillance and coordination. Risk communication is a governance approach with three strategic key activity areas: information (gathering, assessing, and sharing), communication (methods, strategy, and key contents), and coordination (across different administrative levels) (13). These risk communication activities are closely linked with the notification and reporting requirements introduced by IHR (2005). “*This broad notification requirement aims at detecting, early on, all public health events that could have serious and international consequences, and preventing or containing them at source through an adapted response before they spread across borders*” (14). These risk communication categories (information, communication, and coordination) are the main pillars that can inform and facilitate the surveillance process, outbreak response, and outbreak management.

## A Model for Earlier Detection

To elucidate the outbreak detection process, we developed a generic five-step model of disease detection, starting with a health “event” and ending with outbreak realization at a higher or national level. The central concept behind this model is to delineate the steps in outbreak detection, in order to provide a platform upon which to investigate what the conditions are for each step and the factors that facilitate or block these conditions (see Table 1).

**Table 1:** Five-step detection model with conditions and influencing factors.

**Event**	**Conditions**	**Facilitating Factors**	**Blocking Factors**
Step 1 Recognition			
Step 2 Local reporting			
Step 3 Local assessment			
Step 4 Higher-level reporting			
Step 5 Higher-level assessment			

### Five-Step Generic Detection Model

The five steps are:

Step 1: Local recognition—that there is “something” going on that is unusual, strange, worrying, etc.
Step 2: Local reporting.
Step 3: Local assessment.
Step 4: Higher-level reporting.
Step 5: Higher-level assessment and outbreak realization.

### Workshop Methodology

For each of the five steps in the detection model, there is a set of conditions that needs to be in place. To gather, identify, and assess key conditions, information and insight is needed to provide this input. This data is collected by applying a systematic methodology working with small, facilitated groups using analytical templates and matrices (see description of the “incubator approach” in Box 1).

Box 1 **Incubator approach.**

**Incubator approach**

The incubator approach is a novel methodology developed by dickmann risk communication drc| to gather and analyze social or health system-related information. This approach builds on a variety of social science theories [e.g., positive deviance (15–17) and behavioral economics (e.g., decision making in groups)] and provides a structured, systematic, interactive, and collaborative process to gather and assess qualitative, context-sensitive information. Central to this approach is intense, interactive work with a group of stakeholders applying a logical flow in a series of smaller, facilitated working groups and plenary discussions. Participants are stakeholders representing a large range of perspectives, sectors, and disciplines. The group work uses analytical templates and matrices to systematically deconstruct and disrupt current understanding and concepts and rebuild and create interventions to improve the situation (e.g., *earlier* detection).

Outputs of these incubators are locally informed, structured insights that can be used to design interventions, monitor progress, and measure outcomes and impact.

More on https://www.dickmann-drc.com.

Each condition has facilitating and blocking factors. Working through scenarios in small working groups, this information is disentangled in a group process.

In a quantitative analysis, the most influential (“priority”) facilitating and blocking factors can be identified assuming that the most important factors are those that are provided the most frequent in the group decision-making process.

These priority factors are the targets of interventions that aim to accelerate detection. In a modified theory of change approach, these interventions are designed in a moderated group process (see Table 2).

**Table 2:** Modified theory of change approach and interventions.

	**Current Situation**	**Desired Situation**	**Indicators of Change**	**Interventions**
(1). Local Recognition				
(2). Local Reporting				
(3). Local Assessment				
(4). Higher-level Reporting				
(5). Higher-level Assessment				

## Data Analysis

The workshop methodology (incubator approach, Box 1) was employed in two workshops encompassing four regional infectious disease surveillance networks (see Box 2):

- Two networks in Southeast Europe and the Middle East: Southeast European Center for Surveillance and Control of Infectious Diseases (SECID) and Middle East Consortium on Infectious Disease Surveillance (MECIDS); and with
- Two networks in Southern Africa: Southern African Centre for Infectious Disease Surveillance (SACIDS) and East African Integrated Disease Surveillance Network (EAIDSNet).

Box 2 **Workshop participants.**

**Participants**

Two workshops were held in July 2015. The first was for the SECID and MECIDS networks in Budva, Montenegro. The second was for the SACIDS and EAIDSNet networks in Dar es Salaam, Tanzania. Participants (*n* = 33) at the Budva workshop were IHR focal points from Albania (3), Bosnia and Herzegovina (4), Bulgaria (3), Croatia (3), Israel (2), Kosovo* (3), Macedonia (3), Montenegro (2), Moldova (3), Romania (1), Serbia (3), and Slovenia (2) with experience with or responsibility for the management of emerging health threats and the authority to induce change in their countries (senior level, e.g., head of unit or director).

SECIDS: www.secids.com

MECIDS: www.mecidsnetwork.org

Participants (*n* = 19) at the Dar es Salaam workshop were senior “shapers” in their countries with experience or responsibility for the management of emerging health threats and the authority to induce change in their countries (senior level, e.g., director) coming from Zambia (4), Tanzania (12), Uganda (1), and Kenya (2).

SACIDS: www.sacids.org

EAIDSNet: www.eac.int/sectors/health/disease-prevention-and-control/eaidsnet

All networks: www.cordsnetwork.org

*This designation is without prejudice to positions on status and is in line with UNSCR 1244 and the ICJ Opinion on the Kosovo declaration of independence.

Applying the incubator approach (Box 1), we introduced the detection model as a conceptual framework for the workshop and a sorting structure. Participants were divided into smaller working groups to focus on different event types such as human health, animal health, or data (e.g., information via a news provider). Participants used the detection model to conceptualize and identify the following for earlier disease detection for each of these event types:

- Disease detection information, communication, and coordination activities in an ideal scenario for each of the five steps of the detection model;
- Necessary conditions for the activities to take place; and
- Factors that influence whether or not the conditions are in place.

Results of the working groups from each event type (human and animal health and data) were presented and discussed in plenary sessions. At the end of the 2-day workshops, we had empirically identified the most important conditions and factors that influence earlier disease detection within each step of the detection model for each event type, arising out of the interaction (see Box 3).

Box 3 **Data analysis.**

**Data analysis**

1. Coding and categorization by two independent researchers

Two CORDS researchers independently reviewed and categorized the conditions and influencing factors identified at each workshop. The researchers discussed and agreed on final categorization. In some cases, multiple final category tags were assigned to influencing factors based on joint discussion.

2. Priority list of conditions and factors

Conditions and influencing factor categories were then quantitatively ranked in order of importance, based on count within each step of the disease model. The priority influencing factor list includes the three highest-ranking facilitating and blocking factors at each step in the detection model. More than three facilitating and blocking factors were included if there was a tie for the third highest-ranking factor.

3. Feedback on prioritized factors by semi-structured interviews with selected participants

We did a quality check with five select participants from each workshop on the priority influencing factors. Participants provided feedback via semi-structured phone interviews on the final list of priority influencing factors and context-specific indicators of the factors. Three CORDS researchers discussed interview results to determine how to integrate feedback into the framework. In some cases, this resulted in inclusion of more than the top three most important factors within a step in the detection model. We assumed that factors added by interviewees are the least important within the detection model step unless indicated otherwise during the interviews.

## Patient and Public Involvement Statement

The workshop methodology does not address patients directly, yet participants of the workshops came from a broad range of professions (e.g., medical doctors, journalists, and community care workers) representing cultural and social diversity.

## Results

### Necessary Conditions

To compare the most important necessary conditions for earlier detection, we present a matrix of necessary condition domains in each region (see detailed list in Table 3). The necessary condition domains are based on the following categories that were used during data collection at the incubator workshops: governance, technical capacity, human resource capacity, knowledge, skills, and attitudes/beliefs.

**Table 3:** Relative importance of necessary condition domains within each step in the detection model.

**MECIDS/SECID**	**Governance policy**	**Capacity technical**	**Capacity HR**	**Competence knowledge**	**Competence skills**	**Attitude belief**
(1). Recognition						
(2). Reporting						
(3). Assessment						
(4). Reporting						
(5). Assessment						
*Overall*	1 (most)	3	2	5	4	6 (least)
**EAIDSNet/SACIDS**						
(1). Recognition						
(2). Reporting						
(3). Assessment						
(4). Reporting						
(5). Assessment						
Overall	1 (most)	2	4	3	4	5 (least)

*A scale of light to dark indicates the relative importance of necessary conditions within each step of the detection model for each region. Light-colored cells are the least important, and dark-colored cells are the most important*.

Governance emerged as the most important necessary condition for earlier detection in both regions overall. Legislation and standard operating procedures lay the groundwork for all aspects of disease detection. It is critical that these governing tools are available, practical, and clear for each step in the detection model. Attitudes and beliefs emerged as the most subjective and least important condition for earlier detection across the regions. The remaining four categories had different relative levels of importance across the two regions.

In a low-income setting, the most important condition at the early stages of outbreak detection (stages 1–3: recognition, reporting, and assessment) was technical capacity followed and accompanied by governance and policy. In a mid-income setting, the most relevant condition was governance followed by technical and HR capacities.

At higher levels and later stages of outbreak detection (stages 4–5), the most important condition in both regions was governance.

### Priority Factors That Influence Earlier Detection

Influencing factors for earlier detection can, in both regions, be categorized along the main activities of risk communication: information (gathering, assessing, and sharing), communication (methods, strategy, and key contents), and coordination (across different administrative levels), embedded in a politically supportive governance approach with a particular emphasis on local level and local and regional activities (for a detailed list see Table 3). While facilitating factors refer to functional coordination, leadership, communication, and information sharing, blocking factors mainly describe the lack of these (facilitating factors).

At the early local stages of outbreak detection (stages 1–3: recognition, reporting, and assessment), the most important facilitating factors in both regions are coordination at local and regional levels, policy and leadership, and access to information and previous experience. Blocking factors at this local level are in both regions lack of knowledge and motivation, lack of communication, lack of information and diagnostic infrastructure, lack of policy, and disincentives to report.

At higher levels and later stages of outbreak detection (stages 4–5), key facilitating factors are again coordination, communication, and a policy framework, followed by training, tools, and access to information. Blocking factors in both regions at these stages are lack of policy, disincentives, lack of information and information technologies, and poor communication.

## Discussion

### Conditions and Factors

In both low- and mid-income countries, governance was identified as the most important condition allowing earlier detection of infectious disease outbreaks. This comes as a surprise, because many initiatives focus on technical or human resource capacity building, but little attention has been paid to intervening in governance to date. Governance refers to implicit or explicit political, social, religious, cultural, and scientific norms. Governance is an approach and “management” tool; it is the spirit that leads people, who feel that there is something unusual, to do something about it, e.g., to report to a health worker. Factors influencing this condition include awareness, decision-making, communication, coordination, and other social or scientific norms. In order to improve surveillance to detect outbreaks earlier, functional risk communication activities such as coordination, communication, and information sharing, along with leadership, play an important role. These factors ensure that the most important condition, governance, is guiding detection efforts. Facilitating factors during the early stages of outbreak detection at the local level are coordination at local and regional levels, policy and leadership, and access to information and previous experience. The blocks in the early stages are seen in a lack of those facilitating factors. The technical capacity at a local level plays a role, in particular in low-income countries, but is not the most important factor.

In order to improve detection, the focus on technical infrastructure, as often seen in development and capacity-building programs, is probably overstated. In fact, policy and governance activities along with risk communication might be more important targets for strengthening earlier detection.

### Interventions and Measurement

By better understanding the influencing conditions and factors that can lead to earlier detection, interventions can be guided more efficiently and effectively. Both workshops resulted in a list of interventions for future implementation. This systematic framework could also be used for future monitoring and measurement of changes and improvements.

An interesting question to pose would be where should the most effective interventions be directed: following the logic of earlier outbreak detection, it would be at the local level and its early stages of detection (local recognition, reporting, and assessment). However, it could also be that in order to strengthen local recognition, reporting, and assessment, interventions at regional and national levels are more powerful and interventions to strengthen higher-level reporting and assessment at regional and national levels result in earlier detection. Further research needs to be undertaken to complement the evidence using the same methodological framework, possibly including high-income countries, and implementation and testing of interventions could provide more insight into these.

### Limitations

The empirical data on conditions and influencing factors of earlier detection is based on a small sample that needs testing and validation. It applies a generic framework and novel, systematic data collection methodology that, too, requires further application and reflection. It is, however, a systematic approach to gain more qualitative insights about how to reduce time to detection—and not just record a quantitative measurement.

### Perspective

Building on this, we will need to test and validate the key drivers of earlier detection. We propose the following next steps:

1. Develop a measurement framework with indicators summarized in a scorecard, to allow benchmarking and continuous monitoring following the application of the framework;
2. Design and implement intervention studies to test the validity of the indicators, and if targeting the influencing factors identified (by removing blocking factors and/or amplifying facilitating factors) leads to earlier detection.
3. Complement the database with data from high-income countries and compare findings.

## Conclusion

Governance was identified as the most important condition allowing earlier detection of infectious disease outbreaks. This came as a surprise, because many initiatives focus on technical or human resource capacity building, but little attention has been paid to intervening in governance to date. Key influencing factors are risk communication activities and policy at local, regional, and national levels. Interventions targeting the strengthening of risk communication activities at a local level seem a good starting point to test the model and monitor progress.

This research introduced a generic disease detection model and a novel methodology to collect and structure social information. It was applied in low- and mid-income countries. These innovations (model and methodology) can be further used to complement existing evidence and include data from high-income countries and to develop a monitoring framework to assess and evaluate progress.

## Data Availability Statement

All datasets generated for this study are included in the article/supplementary material.

## Ethics Statement

The Jena University Hospital Ethics Committee waived the requirement for ethical approval for this study in accordance with the national legislation and the institutional requirements. This study is a method development and not an experimental setting.

## Author Contributions

PD designed the research concept and crafted the workshop material. LS, EO, and PD carried out the data collection and analysis. LS and PD prepared the draft manuscript that EO, SB, SS, and JL reviewed thoroughly. All authors approved the final version.

## Conflict of Interest

PD is the founder and managing director of the company Dickmann Risk Communication Drc. The remaining authors declare that the research was conducted in the absence of any commercial or financial relationships that could be construed as a potential conflict of interest.
